# The association of cancer-related fatigue on the social, vocational and healthcare-related dimensions of cancer survivorship

**DOI:** 10.1007/s11764-023-01451-9

**Published:** 2023-08-30

**Authors:** Jennifer M. Jones, Doris Howell, Christopher Longo, Karin Olson, Philippe Bedard, Eitan Amir, Shiyu Zheng, Brittany Chow, Lisa Avery

**Affiliations:** 1 Cancer Rehabilitation and Survivorship Program, Princess Margaret Cancer Centre and Department of Psychiatry, University of Toronto, 200 Elizabeth Street, B-PMB-045, Toronto, ON M5G 2C4 Canada; 2 Department of Supportive Care, Princess Margaret Cancer Centre and Lawrence Bloomberg Faculty of Nursing, University of Toronto, Toronto, Canada; 3 Health Policy and Management, DeGroote School of Business, McMaster University, Hamilton, Canada; 4 Faculty of Nursing, University of Alberta, and Edmonton Clinic Health Academy, Edmonton, Canada; 5 Division of Medical Oncology and Hematology, Princess Margaret Cancer Centre and Department of Medicine, University of Toronto, Toronto, Canada; 6 MD Program, University of Toronto, Toronto, Canada; 7 Department of Family and Community Medicine, University of Toronto, Toronto, Canada; 8 Department of Biostatistics, Princess Margaret Cancer Centre, and Dalla Lana School of Public Health, University of Toronto, Toronto, Canada

**Keywords:** Cancer-related fatigue, Social function, Vocational function, Health-care utilization, Breast cancer, Colorectal cancer

## Abstract

**Background:**

Cancer-related fatigue (CRF) is well documented in cancer survivors, but little is known about the personal and societal impact of CRF. This study aimed to examine the impact of CRF in relation to social and vocational functioning and health care utilization in a large sample of post-treatment cancer survivors.

**Methods:**

We conducted a cross-sectional descriptive study of early stage breast and colorectal cancer survivors (*n* = 454) who were within 5 years from treatment completion. Social difficulties (SDI-21), work status, absenteeism and presenteeism (WHO-HPQ) and healthcare utilization (HSUQ) were compared in those with (CFR +) and without (CRF −) clinically significant fatigue (FACT-F ≤ 34).

**Results:**

A total of 32% met the cut-off criteria for CRF (≤ 34). Participants with CRF + had significantly higher scores on the SDI-21 across all domains and 55% of CRF + vs. 11% in CRF − was above the SDI cut-off (> 10) for significant social difficulties. Participants with CRF + were 2.74 times more likely to be unemployed or on leave (95% CI 1.62, 4.61, *p* < 0.001). In the subgroup of participants who were currently working (*n* = 249), those with CRF + reported working on average 27.4 fewer hours in the previous 4 weeks compared to CRF − (*p* = 0.05), and absolute presenteeism was on average 13% lower in the CRF + group (95% CI 8.0, 18.2, *p* < 0.001). Finally, individuals with CRF + reported significantly more physician (*p* < 0.001), other health care professional (*p* = 0.03) and psychosocial visits (*p* = 0.002) in the past month.

**Conclusions and implications for cancer survivors:**

CRF is associated with substantial disruption in social and work role functioning in the early transitional phase of cancer survivorship. Better management of persistent CRF and funding for the implementation of existing guidelines and recommended evidence-based interventions are urgently needed.

**Supplementary Information:**

The online version contains supplementary material available at 10.1007/s11764-023-01451-9.

## Introduction

In North America alone, there are over 19 million people living with a personal history of cancer [1–4], and this number is expected to grow by 24% over the next decade [3]. With increasing proportions of people now surviving cancer and transitioning into the extended survival phases of cancer care, the long-term effects of cancer and its treatments and previously unrecognised chronic morbidity and related disability are of increasing importance.

Cancer-related fatigue (CRF) [5] has been reported as a prevalent and disabling side effect of cancer treatment [6–12] and has been defined as “a distressing, persistent, subjective sense of physical, emotional and/or cognitive tiredness or exhaustion related to cancer or cancer treatment that is not proportional to recent activity and interferes with usual functioning” [13]. During cancer treatment, CRF is an almost universal symptom [14–17] and 25–40% of post-treatment survivors will experience persistent CRF (> 6 months) up to 10 years post-treatment completion [5, 17–31].

While the primary goal of cancer treatments is to eradicate the underlying disease, the long-term goal is for individuals to regain normalcy and return to pre-morbid social and vocational roles after completing treatment [32, 33]. However, CRF can result in significant disability and may disrupt this reintegration process [26, 28, 30, 34]. Despite this, and the evidence of effective interventions and guidelines to manage CRF [13, 17], it remains a poorly managed problem for both cancer patients and survivors [12, 19, 35–37]. This may be due to several factors. Oncology health care providers (HCP) may not routinely ask about fatigue[38] nor its impact on social functioning or vocational roles, and cancer survivors may not report it because they view it as unavoidable [19, 39]. HCPs may also underestimate the importance of CRF [11, 12, 36, 40] and are not always aware of effective interventions and access to supportive care services to manage CRF can be inconsistent and inaccessible [41–48].

To date, while the prevalence and predictors of CRF have been well documented in the literature, little research has focused on the impact of CRF on outcomes such as social functioning, work outcomes and health care utilization [49–53]. Understanding the personal and societal impact of CRF and its impact on cancer survivors’ reintegration to social and vocational roles can help provide a broader understanding and appreciation of its significance, identify targets and outcomes for interventional research and provide useful data to drive change to health care policy and remuneration systems. This study aimed to examine the impact of CRF in relation to social difficulties, work status, work absence (absenteeism), ability to perform work (presenteeism) and health care utilization in a large sample of post-treatment breast and colorectal cancer survivors. Breast and colorectal cancers were selected as they are highly prevalent cancers that are known to be associated with persistent CRF [26]. We hypothesised that cancer survivors suffering from CRF (CRF +) would have increased difficulty with social and vocational functioning and have higher health care and social service utilization compared to those who do not suffer from CRF (CRF −).

## Methods

### Study design and patient selection

This was a cross-sectional descriptive study conducted at Princess Margaret Cancer Centre and Mount Sinai Hospital in Toronto, Canada. Individuals were eligible if they (1) were within 1–5 years of completing primary treatment for early stage (0–III) breast or colorectal cancer, (2) were not receiving current cancer therapies other than adjuvant endocrine therapy, (3) did not have evidence of recurrent or metastatic disease, (4) spoke and read English and (5) were at least 18 years old. After screening for eligibility, individuals attending follow-up clinic visits were approached to participate. Patients who provided consent to participate were given the questionnaire package to complete and return in-clinic or via pre-paid postage envelope.

This study was reviewed and approved by the University Health Network Research Ethics Board and the Mount Sinai Hospital Research Ethics Board.

### Study assessments

#### Demographic and clinical information

A *Patient Information Questionnaire* was used to assess demographic variables (age, sex, education, income, employment, marital status, ethnicity). Clinical data was extracted from the electronic patient record.

#### Cancer-related fatigue

CRF was assessed using the 13-item *Functional Assessment of Cancer Therapy-Fatigue (FACT-F)* subscale [54]. The FACT-F has been extensively used in a range of cancer populations [55, 56] and correlates well with the International Statistical Classification of Diseases and Related Health Problems (ICD-10) criteria for cancer-related fatigue [18]. Each item is answered on a five-point scale and scores range from 0 (maximum fatigue) to 52 (minimum fatigue) with lower scores indicating higher fatigue. The recommended cut-off score of ≤ 34 on the FACT-F has a sensitivity of 0.91 and a specificity of 0.75 and can accurately predict ICD status for CRF in 93% of patients [57].

#### Social functioning

The *Social Difficulties Inventory* (SDI) is a valid and reliable [58–61] 21-item questionnaire designed to assess social difficulties experienced by cancer patients over the preceding month [60, 62]. The SDI-21 is scored by calculating a 16-item summary score (SD-16), ranging from 0 to 44, which further comprises three factor analysis-derived subscales: Everyday Living, Money Matters and Self-and-Others [59]. A cut-off of ≥ 10 has been recommended to identify patients experiencing social distress and has 80% sensitivity and 75% specificity compared to clinical assessment, and a difference of 2 points on the sub-scales and 3 on the SD-16 are considered to represent a meaningful clinically important difference [59, 63].

#### Work status, absenteeism and presenteeism

Participants were asked about their work status and type of work. Participants who indicated that they were currently employed (full or part-time) or self-employed were then asked to complete the absenteeism and presenteeism questions of the *World Health Organization’s Health and Work Performance Questionnaire short form* (WHO-HPQ) [64, 65]. The WHO HPQ has good validity and reliability [66–69] and provides a measure of absenteeism and presenteeism. Absolute absenteeism focuses on the total number of hours an employee is absent, while relative absenteeism provides a more contextualised view by comparing the absenteeism rate to the expected number of hours by the employer. Absolute absenteeism (AA) is measured in terms of work hours lost in the past 4 weeks and reported in raw hours. A higher score on AA indicates more absenteeism (hours lost). Relative absenteeism (RA) is reported as the percentage of the hours one is expected to work and can range from a negative value (when a person works more than expected) to a maximum of 1.0 (when the person is always absent). Absolute presenteeism (AP) measures self-rated overall job performance on the days worked over the past 4 weeks and has a lower bound of 0 (worst possible performance) and an upper bound of 100 (best possible performance). Presenteeism is a measure of actual performance in relation to possible performance as rated by the individual. In this case, a higher score indicates a lower amount of lost performance. Relative presenteeism (RP) is the ratio of one’s self-rated performance compared to their rating of the performance of most workers at the same job. The distribution of RP is restricted to the range of 0.25 to 2.0, with the lowest score indicating the worst relative performance (25% or less of other workers’ performance), and the highest score the best performance (200% or more of other workers’ performance) [70]. Respondents were also asked to compare their overall job performance over the past 4 weeks with the performance of most other workers in the same job on a 7-point scale from “You were a lot better than others” to “You were a lot worse than others”.

#### Health service utilization

Health Service Utilization was assessed using the *Health Services Utilization Questionnaire* [71, 72] modified for use with a cancer population [73]. Service types are grouped into five categories: Physician visits, Other Health Professionals (nursing, allied health, pharmacist), Hospital visits and services, Psychosocial Services (professional counselling, support group, information supports, financial counselling or assistance programmes, spiritual support) and Home Support (community care access centre services, housekeeping, transportation services, home delivered meals). Participants were asked which of these services they have used in the past month including the frequency of use.

### Statistical analysis

Participant characteristics were described for each cancer site (breast or colorectal). Participants were classified as having CRF if their FACT-F score was $\le 34$ (CRF +). Categorical demographic correlates of CRF were compared with ${\chi }^{2}$ tests and Wilcoxon rank sum test for continuous variables. To assess the association between CRF and social functioning, independent sample *t* tests were conducted to compare mean SDI domain scores (everyday living, money matters and self) and the SDI-16 score between those CRF + and CRF − and presented on box plots to illustrate the distribution of SDI scores. Cohen’s *d* effect sizes were also calculated. Working status was classified into four categories: working (full or part-time), retired, on leave/disability or not in paid employment and compared between those CRF + and CRF − using a ${\chi }^{2}$ test. The odds ratio of working (full or part-time) vs. not working (not working/on leave), excluding retired persons, was also calculated. Comparisons of workplace performance, including AA, RA, AP and RP between the groups, were conducted using independent *t* tests for the sub-sample of patients who reported working full, part-time, or in self-employment (*n* = 228), and Cohen’s *d* effect sizes were calculated. Relative workplace performance scores were collapsed to three levels (better than others, about the same, worse than others), and responses between those CRF + and CRF − were compared using Fisher’s exact test. The use of health care resources, measured as number of visits in the previous month, was compared using independent sample *t* tests, and Cohen’s *d* effect sizes were calculated. For each research question, a Holm’s correction [74] was applied to control for multiple testing, and results were considered statistically significant if the adjusted two-sided *p*-value was < 0.05. Sensitivity analyses were conducted using multivariate regression to determine if age or cancer type influenced the findings as follows: logistic modelling of the risk of CRF + as a function of SDI score, age and cancer type for each SDI domain, the odds of working as a function of CRF + , age and cancer type, and linear modelling of the number of health care visits as a function of CRF + , age and cancer type. Analyses were conducted using R version 4.2.2 [75] and all tests were two-sided.

## Results

A total of 625 eligible patients were approached and 454 consented and returned the completed questionnaire (overall participation rate 73%; breast (*n* = 302) 75% and colorectal (*n* = 152) 68%). Demographic and clinical data are presented in Table 1. Participants were on average 2.4 years from end of treatment (median, 2.2 years; range, 0.2–5.0 years). Just over half the sample (54%) was born in Canada and most were living with a partner (67%).

**Table 1:** Participant characteristics

	Full sample (*n* = 438) *n* (%)	Breast cancer (*n* = 302) *n* (%)	Colorectal cancer (*n* = 136) *n* (%)
Age group			
< 45 yrs	58 (14)	46 (16)	12 (9)
45–54 yrs	135 (32)	101 (34)	34 (26)
55–64 yrs	122 (29)	83 (28)	39 (30)
65–90 yrs	112 (26)	65 (22)	47 (36)
Missing	11	7	4
Sex			
Male	68 (16)	3 (1)	65 (49)
Female	361 (84)	293 (99)	68 (51)
Missing	9	6	3
Marital status			
Married/life partner	287 (67)	201 (68)	86 (65)
Divorced/separated	58 (14)	35 (12)	23 (17)
Widowed	27 (6)	19 (6)	8 (6)
Single/never married	56 (13)	40 (14)	16 (12)
Missing	10	7	3
Working status			
Currently working	249 (57)	180 (60)	69 (51)
On leave/disability	29 (7)	21 (7)	8 (6)
Retired	111 (25)	67 (22)	44 (32)
Not working	49 (11)	34 (11)	15 (11)
Place of birth			
Canada	233 (54)	168 (57)	65 (49)
Other	195 (46)	128 (43)	67 (51)
Missing	10	6	4
Household income (CDN dollars)			
Below 30,000	71 (18)	47 (17)	24 (20)
30,000–60,999	84 (21)	58 (21)	26 (21)
61,000–90,000	78 (20)	55 (20)	23 (19)
Over 90,000	160 (41)	112 (41)	48 (40)
Missing	45	30	15
Years since diagnosis			
Mean (sd)	3.1 (1.4)	3.1 (1.5)	3.0 (1.2)
Median (min, max)	2.8 (1.1, 19.2)	2.8 (1.1, 19.2)	2.7 (1.2, 6.1)
Missing	16	6	10
Years since last treatment			
Mean (sd)	2.3 (1.1)	2.4 (1.1)	2.3 (1.1)
Median (min, max)	2.2 (0.2, 5.0)	2.2 (0.9, 5.0)	2.2 (0.2, 4.9)
Missing	6	0	6
Received surgery			
No	51 (12)	25 (8)	26 (19)
Yes	386 (88)	277 (92)	109 (81)
Missing	1	0	1
Received chemo			
No	106 (24)	86 (28)	20 (15)
Yes	331 (76)	216 (72)	115 (85)
Missing	1	0	1
Received radiation			
No	103 (24)	56 (19)	47 (35)
Yes	334 (76)	246 (81)	88 (65)
Missing	1	0	1

Of the 454 participants, the mean (sd) score on the FACT-F was 38.0 ± 11.5, and 147 (32%) met the cut-off criteria for CRF (≤ 34). No demographic or clinical variables were significantly associated with the presence of CRF.

### Social functioning

**Fig. 1:**
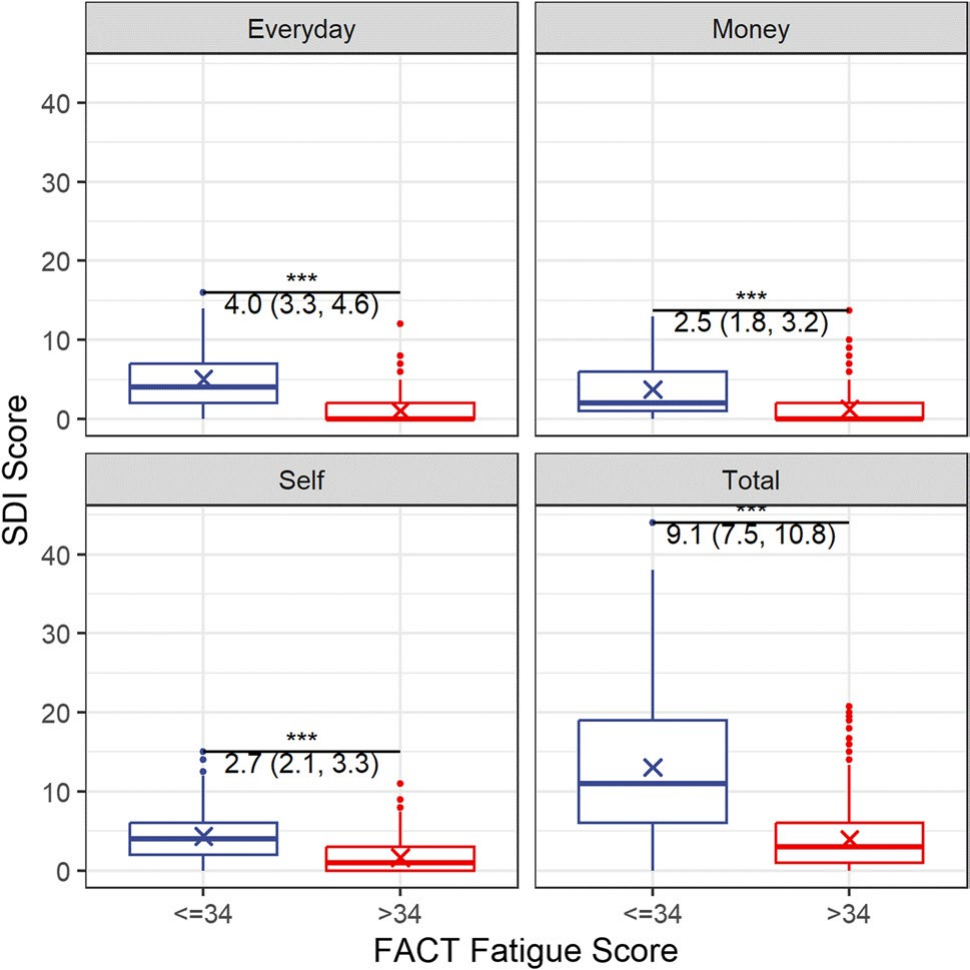
Comparisons of Social Difficulties Index scores for those with and without CRF. Means for each group are marked with an “x” and mean differences with 95% CI are presented for each SDI domain. All *p*-values are < 0.001 after the application of Holm’s correction to control for multiple comparisons

CRF + was associated significantly with higher scores on the SDI across all domains (Fig. 1): everyday living (5.0 ± 3.9 vs. 1.1 ± 1.7, *p* < 0.001, Cohen’s *d* 1.13), money matters (3.6 ± 3.9 vs. 1.3 ± 1.9, *p* < 0.001, Cohen’s *d* 0.68), self (4.3 ± 3.3 vs. 1.6 ± 1.9, *p* < 0.001, Cohen’s *d* 0.87) and on overall social distress (SD-16) (12.8 ± 9.5 vs. 4.0 ± 4.2, *p* < 0.001, Cohen’s *d* 1.04). Fifty-five percent of individuals’ CRF + and 11% of those CRF − were above the SDI cut-off (> 10) for significant social difficulties.

### Work status and performance

**Fig. 2:**
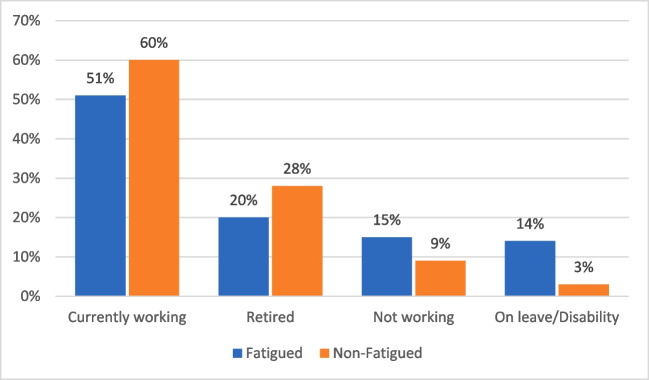
Distribution of working status for those with and without fatigue

Participants with CRF + had significantly different work profiles than those CRF − (Fig. 2) and more likely to be not working or on leave/disability (OR = 2.72 95% CI 1.62, 4.61, *p* < 0.001).

In the subgroup of participants who were currently working (*n* = 249), those who were CRF + reported working on average 27.4 fewer hours in the previous 4 weeks vs. those who were CRF − (95% CI 5.3, 49.4, *p* = 0.05) (Table 2). There was no significant difference in absolute or relative absenteeism scores. However, absolute presenteeism for those CRF + was on average 13% lower (95% CI 8.0, 18.2, *p* < 0.001). Furthermore, those with CRF + reported a greater discrepancy between themselves and other workers in their jobs (relative presenteeism), than those CRF − (95% CI 0.1, 0.2, *p* < 0.001).

**Table 2:** Differences in work-related performance for those with fatigue and without fatigue

Past 4 weeks	CRF + (*n* = 72) Mean (sd)	CRF − (*n* = 177) Mean (sd)	Difference (95% CI)	Cohen’s *d*	*p*-value*
Hours worked	126.7 (85.7)	154.1 (61.5)	27.4 (5.3, 49.4)	0.31	0.05
Absolute absenteeism	4.7 (50.4)	− 5.4 (38.0)	− 10.1 (− 23.2, 2.9)	0.19	0.25
Relative absenteeism	0.0 (0.4)	0.0 (0.2)	− 0.1 (− 0.2, 0.0)	0.19	0.25
Absolute presenteeism	70.5 (17.8)	83.6 (14.0)	13.1 (8.0, 18.2)	0.65	** < 0.001**
Relative presenteeism	0.9 (0.2)	1.1 (0.2)	0.1 (0.1, 0.2)	0.52	** < 0.001**

*The p-values were adjusted to control for multiple comparisons

**Fig. 3:**
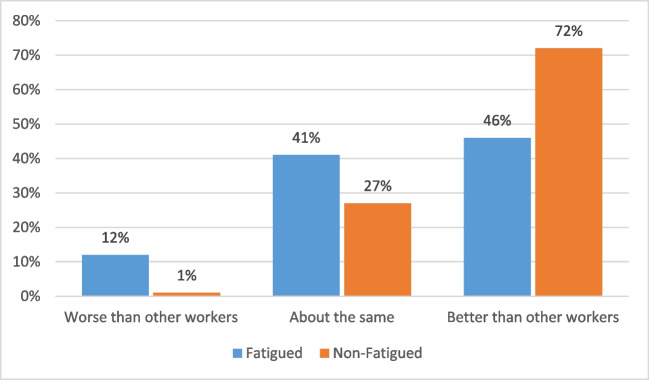
Relative performance as a proportion of those in the fatigued and not fatigued groups

Reported relative performance of workers with and without fatigue was also compared, and there was a significant difference in the proportion of participants with fatigue who rated their work performance as better vs. worse than their co-workers (Fig. 3) (*p* < 0.001).

### Health care use

**Fig. 4:**
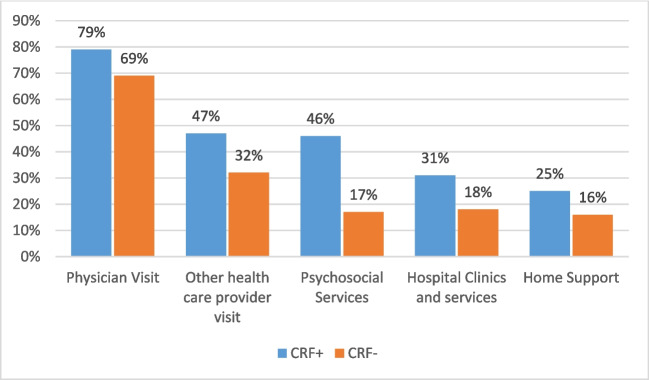
Proportion of participants accessing health care service over the past month (fatigued and not fatigued groups)

The proportion of participants with and without fatigue who reported accessing services is shown in Fig. 4.

Those with fatigue reported significantly more physician, other health care professional and psychosocial visits (Table 3).

**Table 3:** Differences in health care use, measured as the number of visits in the preceding 4 weeks, for those with and without fatigue

Variable	CRF + (*n* = 142) Mean (sd)	CRF − (*n* = 296) Mean (sd)	Difference (95% CI)	Cohen’s *d*	*p*-value*
Physician visits	2.1 (2.1)	1.3 (1.4)	0.8 (0.4, 1.2)	0.41	** < 0.001**
Other health care professional visits	1.4 (2.5)	0.8 (1.9)	0.6 (0.2, 1.1)	0.25	**0.03**
Hospital clinics and services	0.5 (1.2)	0.2 (0.8)	0.2 (0.0, 0.5)	0.20	0.07
Psychosocial services	1.1 (2.3)	0.4 (1.5)	0.8 (0.3, 1.2)	0.34	**0.002**
Home support	1.0 (2.7)	0.5 (1.6)	0.5 (0.0, 1.0)	0.19	0.07

*The *p*-values were adjusted to control for multiple comparisons

### Effect of age and cancer type

The results of the sensitivity analyses are reported in the supplemental data. Controlling for age and cancer type did not modify the relationship between CRF and social function (Table S1), work status (Table S2), workplace performance (Table S3), nor health care use (Table S4). Neither age nor cancer type was significant predictors in any of the sensitivity analyses with the exception that, on average, an extra year of age was associated with 1.7 fewer hours worked in the previous 4 weeks (Table S4).

## Discussion

This study provides a detailed examination of the association between CRF and social and vocational functioning, and health care utilization in a large sample of post-treatment cancer survivors using a validated fatigue scale. The findings demonstrate that cancer survivors with CRF experience challenges to reintegration including social difficulties and problems with work attendance and work performance. Furthermore, cancer survivors with CRF report more health care utilization over the past month with higher rates of health care provider visits and hospital visits, and more psychosocial support visits. This is important information that suggests a significant personal and societal impact of CRF and can be helpful evidence to advocate for the funding of the implementation of existing evidence-based guidelines and the services and interventions recommended. Furthermore, given the association with CRF, social and vocational functioning and health care utilization can be important outcomes to measure in future interventional studies addressing CRF.

The post-treatment transitional phase of cancer survivorship has been described as one of the most stressful times for survivors, and they describe needing support and guidance as to how to recover their health and to re-engage in their social and work-life roles [76] . Social functioning is an important reintegration target, and social connections have been shown to be beneficial to overall physical health, well-being and longevity [77–80], as well as better cancer survival and a lower risk of cancer mortality [81–84]. To date, deficits in social roles and activities have been reported in cancer survivors [85, 86], but little research has examined the related factors. One study examining social functioning in cancer survivors reported a negative association between social activities and CRF, though this was not the primary focus of the study [50]. In our current study, we found a difference of almost 9 points between those with and without CRF on the SDI-16, which has a minimal clinically important difference of 3, and 55% of individuals with CRF met cut-off for clinically significant social difficulties.

In terms of work and work function, we found CRF was associated with a 2.72 times higher odds of being unemployed or on leave. Previous studies have shown that cancer survivors experience higher rates of unemployment compared to non-cancer peers [87, 88], and fatigue and exhaustion are barriers to return to work [89, 90]. Unemployment can result in significant wage loss and financial burden and can impact quality of life [91, 92]. In some situations, survivors must return to work despite physical limitations due to lack of support and financial and medical insecurity [93, 94]. In those who were currently working, individuals with CRF + worked fewer hours over the prior 4 weeks (− 27.4) compared to those who were CRF − , and there was a difference in work performance (presenteeism). Presenteeism, along with absenteeism, can impact earnings, and individuals experiencing dysfunctional presenteeism have lower earnings on average [95] and an increased risk for financial toxicity [96]. Along with the personal impact, there is a significant societal and economic cost of not returning to work and not performing at work [97, 98]. The National Institute of Health in the USA estimated that in 2010, the cost of lost productivity accounted for 61% of the total cost of cancer, compared to 39% for the direct costs related to treatment [99]. Furthermore, estimates of the cost of illness to businesses have reported that impaired presenteeism results in significantly more costs compared to absenteeism [100, 101]. Taken together, these findings highlight the need to improve employment rates and job performance among cancer survivors with fatigue. Interestingly, the effects of interventions to facilitate return to work and work performance, which have primarily targeted work-related factors, have not been effective [102, 103]. This is likely because health-related factors such as persistent fatigue, which are associated with work status and productivity factors, have not been targeted. Moving forward, multifaceted interventions targeting return to work and work function must also consider CRF as a potential mediator and target for intervention [104, 105]. For example, exercise interventions, which are known to effectively reduce cancer-related fatigue [106, 107], have also been shown to promote return to work and reduce missed work hours [108, 109] and are more effective than occupational support or counselling interventions alone [110, 111].

Finally, we found that cancer survivors with CRF had more visits to physicians and other health care professionals (i.e. in-home nursing care, physiotherapists, pharmacist) and accessed more psychosocial services (i.e. social worker, psychologist, support group, financial counselling). Increased health care utilization has been reported in post-treatment cancer survivors compared to age-matched controls [112–115], and fatigue has been reported as one of the most common presenting complaints. While future research using proper health costing estimates are required, it is clear that CRF results in a significant cost to our health care system.

Based on a growing body of intervention research, guidelines for the management of CRF have been developed and adopted by cancer organisations [13, 17, 116, 117]. Exercise has the strongest evidence of reductions in CRF [118–120]. However, simply recommending that people with CRF exercise is likely not effective, and CRF can be a significant barrier to participation in physical activity [121, 122]. In the presence of clinically significant fatigue, current guidelines suggest referral to rehabilitation or exercise specialist for a supervised cancer exercise programme [123]. This has led to recommendations to include exercise science professionals and implement exercise-based rehabilitation as an integral part of cancer care [124, 125]. These programmes can help to address fatigue and optimise physical functioning so that survivors can engage in activities of daily living and participate in the broader community [44]. Despite this, oncology rehabilitation and cancer-specific exercise services have been omitted from large-scale cancer initiatives, and government health care funding dedicated to cancer rehabilitation remains limited in many countries including Canada [41, 45, 46, 124].

The results of the present study should be interpreted within the context of its limitations. This study was conducted in a large urban centre in a high-income country, and the labour market conditions and cultural values are not representative of all settings. This was cross-sectional and therefore the nature of these relationships cannot be determined. While we obtained a good response rate of 73%, there is a possibility of non-response bias. Due to ethical restrictions, data on the non-responders was not available, though previous work by our group with this population found no differences between responders and non-responders based on age, stage of disease, treatments received, or current hormone therapy [26], and work from other groups has shown non-responders may not systematically differ from responders on important baseline variables [126]. Additionally, it is important to note that CRF often co-occurs or clusters with other symptoms such as pain, insomnia and mood disturbances [127], which were not measured in the current study and may mediate or moderate the relationship between CRF and our measured outcomes. Future studies are needed to longitudinally examine the differential role of these symptoms overtime. Finally, the study sample was restricted to breast and colorectal cancer survivors within 5 years of treatment completion. Future research should examine these outcomes in long-term survivor populations to examine if the impact of CRF diminishes as individuals adapt. Future studies should also assess the economic burden of CRF including the burden on the healthcare system, society and those individuals with CRF and their caregivers. Furthermore, studies are needed to demonstrate the benefits and costs of different interventions and clinical services, as this has greater value to policymakers than efficacy of a programme without consideration of its feasibility or the costs of delivery [128].

Despite the limitations, this is the first study to examine the impact of CRF on social and vocational functioning and health care utilization in a large sample of post-treatment cancer survivors using validated tools. Although CRF is a prevalent and consequential symptom and there are guidelines on its detection and management, CRF remains poorly managed. The results from this study provide evidence on the substantial disruptive impact of CRF on the lives of cancer survivors and suggest broader social and financial repercussions. Funding of the implementation of existing guidelines and the recommended evidence-based interventions is urgently needed.

## Supplementary Information

Below is the link to the electronic supplementary material.

Supplementary file1 (DOCX 23 KB)

## Data Availability

Due to REB restrictions, we are not permitted to upload data to a public data repository. However, we can make data available upon request with a data transfer agreement.
