# Supplementary material for: The association of cancer-related fatigue on the social, vocational and healthcare-related dimensions of cancer survivorship
Source: J Cancer Surviv. 2023 Aug 30;19(1):97–108. doi: 10.1007/s11764-023-01451-9 (PMC11813975; doi:10.1007/s11764-023-01451-9)
Supplement: Supplementary file 1 — Supplementary file1 (DOCX 23 KB) [file 11764_2023_1451_MOESM1_ESM.docx]

**Sensitivity analysis of relationship between social function and CRF+**

Table S1: Results of separate multivariable models estimating the odds of cancer-related fatigue for each SDI score, controlling for age and cancer type. Unit increases in SDI scores are associated with significantly increased odds of CRF (p<0.001, all variables after Holm’s correction), while age and cancer type were not

| **SDI Score** | **OR(95%CI)** | **Age** | **Breast Cancer** |
| --- | --- | --- | --- |
| Everyday | 1.82 (1.60, 2.06) | 0.98 (0.96, 1.01) | 1.51 (0.84, 2.72) |
| Money | 1.35 (1.24, 1.47) | 1.00 (0.98, 1.02) | 1.46 (0.88, 2.41) |
| Self | 1.56 (1.40, 1.73) | 1.01 (0.99, 1.03) | 1.49 (0.88, 2.52) |
| Total | 1.25 (1.19, 1.31) | 1.01 (0.98, 1.03) | 1.51 (0.86, 2.66) |

**Sensitivity analysis of differences in work status for those CRF+ vs CRF-**

*Table S2: Odds of being in work for CRF+ vs CRF- controlling for age and cancer type.*

|  | OR(95%CI) | p-value |
| --- | --- | --- |
| **FACT_Fatigue** |  | **<0.001** |
| Not Fatigued | Reference |  |
| Fatigued | 2.83 (1.65, 4.88) |  |
| **Age** | 1.00 (0.97, 1.03) | 0.79 |
| **Diagnosis** |  | 0.50 |
| Colorectal Cancer | Reference |  |
| Breast Cancer | 0.81 (0.44, 1.49) |  |

**Sensitivity analysis of differences in work place performance for those CRF+ vs CRF-**

Table S3: Results of separate multivariable models estimating the differences in work related performance for those without fatigue controlling for age and cancer type. P-values have been adjusted with a Holm’s correction.

| **Past 4 weeks** | Not Fatigued | Age | Breast Cancer |
| --- | --- | --- | --- |
| Hours Worked | 25.35 (6.30, 44.41)  p=0.11 | -1.67 (-2.63, -0.71)  **p=0.009** | -17.70 (-36.97, 1.57)  p=0.75 |
| Absolute Absenteeism | -11.04 (-22.93, 0.85)  p=0.75 | 0.54 (-0.06, 1.14)  p=0.75 | 1.68 (-10.34, 13.70)  p=1.00 |
| Relative Absenteeism | -0.07 (-0.15, 7.3e-03)  p=0.75 | 2.8e-03 (-1.2e-03, 6.8e-03)  p=1.00 | 0.02 (-0.07, 0.10)  p=1.00 |
| Absolute Presenteeism | 14.04 (9.40, 18.69)  p<0.001 | -0.11 (-0.34, 0.12)  p=1.00 | 0.91 (-3.74, 5.56)  p=1.00 |
| Relative Presenteeism | 0.14 (0.09, 0.20)  p<0.001 | -2.5e-03 (-5.4e-03, 4.7e-04)  p=0.75 | -1.4e-03 (-0.06, 0.06)  p=1.00 |

**Sensitivity analysis of differences in health care use for those CRF+ vs CRF-**

Table S4: Results of separate multivariable models estimating the increase in health care use controlling for age and cancer type. Patients with fatigue had significantly greater health care use than those without fatigue (p<0.001, all variables), neither age nor cancer type are associated with increased use. Adjusted difference represents the estimated number of extra visits a patient with fatigue would have in the preceding four weeks, controlling for age and type of cancer.

| Preceding 4 weeks | Adjusted Difference | Age | Breast Cancer |
| --- | --- | --- | --- |
| **Physician visits** | 0.91 (0.57, 1.25) | -7.5e-03 (-0.02, 6.6e-03) | -0.19 (-0.53, 0.16) |
| **Other Health Care Professional visits** | 0.66 (0.22, 1.10) | -8.9e-03 (-0.03, 9.3e-03) | 0.14 (-0.31, 0.58) |
| **Hospital clinics and services** | 0.26 (0.07, 0.46) | 1.8e-03 (-6.3e-03, 9.9e-03) | -0.13 (-0.33, 0.06) |
| **Psychosocial services** | 0.74 (0.37, 1.11) | -6.8e-03 (-0.02, 8.5e-03) | 0.36 (-0.02, 0.73) |
| **Home support** | 0.54 (0.12, 0.96) | -3.3e-03 (-0.02, 0.01) | -0.18 (-0.61, 0.24) |
